# Supplementary material for: Geo-epidemiological risk stratification to select malaria interventions, case of Mali
Source: PLOS Glob Public Health. 2025 Nov 7;5(11):e0004796. doi: 10.1371/journal.pgph.0004796 (PMC12594328; doi:10.1371/journal.pgph.0004796)
Supplement: S1 Table — These data indicate that access to healthcare is poor, except in Bamako. (DOCX) [file pgph.0004796.s003.docx]

S1 Table: Situation of CHWs, population, and accessibility rate by health district

| Region | District | Number of CHWs | Population | Population within 5km | Accessibility in 5km |
| --- | --- | --- | --- | --- | --- |
| Kidal | Abeibara | 20 | 15384 | 3868 | 25.14 |
| Taoudenni | Achouratt | 0 | 37827 | 7124 | 18.83 |
| Gao | Almoustarat | 0 | 28522 | 7834 | 27.47 |
| Taoudenni | Al-Ourche | 0 | 56931 | 8349 | 14.67 |
| Menaka | Anderamboukane | 0 | 28261 | 7085 | 25.07 |
| Gao | Ansongo | 94 | 197058 | 78610 | 39.89 |
| Taoudenni | Arawane | 0 | 31651 | 5199 | 16.43 |
| Kayes | Bafoulabe | 62 | 191910 | 96070 | 50.06 |
| Koulikoro | Banamba | 86 | 284530 | 151068 | 53.09 |
| Mopti | Bandiagara | 58 | 474607 | 227007 | 47.83 |
| Mopti | Bankass | 278 | 393230 | 165171 | 42.00 |
| Segou | Baroueli | 50 | 302959 | 163682 | 54.03 |
| Segou | Bla | 60 | 423643 | 239070 | 56.43 |
| Sikasso | Bougouni | 202 | 692864 | 269206 | 38.85 |
| Taoudenni | Boujbeha | 0 | 35634 | 7385 | 20.72 |
| Gao | Bourem | 0 | 145249 | 46709 | 32.16 |
| Bamako | CI | 0 | 501320 | 501320 | 100.00 |
| Bamako | CII | 0 | 238854 | 238852 | 100.00 |
| Bamako | CIII | 0 | 192620 | 192620 | 100.00 |
| Bamako | CIV | 0 | 448525 | 448525 | 100.00 |
| Bamako | CV | 0 | 619788 | 619788 | 100.00 |
| Bamako | CVI | 250 | 702893 | 702893 | 100.00 |
| Kayes | Diema | 63 | 316259 | 144474 | 45.68 |
| Koulikoro | Dioila | 130 | 396733 | 177692 | 44.79 |
| Tombouctou | Diré | 0 | 164612 | 60607 | 36.82 |
| Mopti | Djenne | 56 | 309365 | 162872 | 52.65 |
| Mopti | Douentza | 56 | 369867 | 141691 | 38.31 |
| Koulikoro | Fana | 147 | 337958 | 197051 | 58.31 |
| Taoudenni | Foum-Alba | 0 | 18648 | 1365 | 7.32 |
| Gao | Gao | 78 | 357720 | 231490 | 64.71 |
| Tombouctou | Goundam | 2 | 222023 | 106341 | 47.90 |
| Tombouctou | Gourma-Rharous | 0 | 164704 | 38366 | 23.29 |
| Menaka | Inekar | 0 | 8096 | 3192 | 39.43 |
| Sikasso | Kadiolo | 74 | 360310 | 236305 | 65.58 |
| Koulikoro | Kalabancoro | 59 | 369697 | 251725 | 68.09 |
| Koulikoro | Kangaba | 67 | 150644 | 106964 | 71.00 |
| Koulikoro | Kati | 99 | 750595 | 477867 | 63.67 |
| Kayes | Kayes | 121 | 766367 | 536571 | 70.01 |
| Kayes | Kenieba | 38 | 294273 | 140942 | 47.89 |
| Kidal | Kidal | 66 | 49486 | 21724 | 43.90 |
| Sikasso | Kignan | 53 | 174479 | 72569 | 41.59 |
| Kayes | Kita | 99 | 516751 | 258365 | 50.00 |
| Koulikoro | Kolokani | 76 | 349867 | 129143 | 36.91 |
| Sikasso | Kolondieba | 141 | 304553 | 110956 | 36.43 |
| Mopti | Koro | 60 | 540252 | 232002 | 42.94 |
| Koulikoro | Koulikoro | 92 | 315742 | 173956 | 55.09 |
| Sikasso | Koutiala | 118 | 864659 | 523776 | 60.58 |
| Segou | Macina | 81 | 352556 | 157200 | 44.59 |
| Segou | Markala | 145 | 328151 | 211569 | 64.47 |
| Menaka | Menaka | 30 | 24918 | 12844 | 51.55 |
| Mopti | Mopti | 81 | 550056 | 346709 | 63.03 |
| Koulikoro | Nara | 57 | 363434 | 146661 | 40.35 |
| Tombouctou | Niafunke | 25 | 272498 | 122265 | 44.87 |
| Sikasso | Niena | 59 | 184983 | 101379 | 54.80 |
| Segou | Niono | 150 | 544896 | 362036 | 66.44 |
| Kayes | Nioro | 59 | 341876 | 211958 | 62.00 |
| Koulikoro | Ouelessebougou | 30 | 297799 | 128711 | 43.22 |
| Kayes | Oussoubidiagna | 23 | 157017 | 79373 | 50.55 |
| Kayes | Sagabari | 5 | 58135 | 28832 | 49.59 |
| Segou | San | 140 | 498215 | 283503 | 56.90 |
| Kayes | Sefeto | 6 | 71053 | 42785 | 60.22 |
| Segou | Segou | 114 | 711423 | 401530 | 56.44 |
| Sikasso | Selingue | 23 | 121991 | 84840 | 69.55 |
| Sikasso | Sikasso | 96 | 731026 | 465594 | 63.69 |
| Taoudenni | Taoudenit | 15 | 26721 | 2693 | 10.08 |
| Mopti | Tenenkou | 38 | 244257 | 121148 | 49.60 |
| Kidal | Tessalit | 4 | 24362 | 7743 | 31.78 |
| Menaka | Tidermene | 0 | 20049 | 4317 | 21.53 |
| Kidal | TinEssako | 0 | 11929 | 813 | 6.82 |
| Tombouctou | Tombouctou | 24 | 184163 | 119369 | 64.82 |
| Segou | Tominian | 63 | 330232 | 118079 | 35.76 |
| Sikasso | Yanfolila | 40 | 194217 | 89598 | 46.13 |
| Kayes | Yelimane | 14 | 263609 | 233087 | 88.42 |
| Sikasso | Yorosso | 59 | 317916 | 169024 | 53.17 |
| Mopti | Youwarou | 51 | 159366 | 55194 | 34.63 |
